# Anxious Individuals Are Impulsive Decision-Makers in the Delay Discounting Task: An ERP Study

**DOI:** 10.3389/fnbeh.2017.00005

**Published:** 2017-01-24

**Authors:** Lisheng Xia, Ruolei Gu, Dandan Zhang, Yuejia Luo

**Affiliations:** ^1^College of Information Engineering, Shenzhen UniversityShenzhen, China; ^2^Key Laboratory of Behavioral Science, Institute of Psychology, Chinese Academy of SciencesBeijing, China; ^3^Institute of Affective and Social Neuroscience, Shenzhen UniversityShenzhen, China; ^4^Department of Psychology, Southern Medical UniversityGuangzhou, China; ^5^Shenzhen Institute of NeuroscienceShenzhen, China

**Keywords:** temporal discounting, anxiety, impulsivity, decision-making, event-related potential

## Abstract

Impulsivity, which is linked to a wide range of psychiatric disorders, is often characterized by a preference for immediate but smaller rewards over delayed but larger rewards. However, debate exists on the relationship between anxiety and impulsivity. Here we use event-related potential (ERP) components as biomarkers in the temporal discounting task to examine the effect of anxiety on inter-temporal decision-making. Our behavioral results indicated that the high trait anxiety (HTA) group made significantly more immediate choices than the low trait anxiety (LTA) group. Compared with the LTA group, shorter response time was associated with immediate rewards in the HTA group. Furthermore, previous studies have demonstrated three ERP components that are associated with impulsivity and/or delay discounting. First, the N1 is an early sensory component involved in selective attention and attention processing for goal-directed actions. Second, the reward positivity (RewP) reflects reward-related dopaminergic activity and encodes reward values. Third, the P3 is regarded as a measure of motivational significance in the decision-making literature. Accordingly, this study found in the immediate-option-evoked ERPs that the HTA group had a larger N1 than the LTA group did. For the delayed-option-evoked ERPs, the HTA group had larger N1 and RewP for the immediate choice than the LTA group did, while the LTA group had a larger P3 for the delayed choice than the HTA group did. These results support the notion that anxiety individuals are impulsive decision-makers in the Delay Discounting Task.

## Introduction

Anxiety, an unpleasant emotional state that often directs an individual's response to threat-related information, plays a significant role in our everyday life (Clark, 1999). Anxiety interrupts daily functions such as attention, working memory, and social skills, resulting in behavioral and cognitive biases (Bishop, 2007). Exploring the cognitive and emotional components associated with anxiety is beneficial for both clinical and non-clinical studies (Grupe and Nitschke, 2013). This study focuses on the relationship between anxiety and impulsive behavior (i.e., a range of non-rational tendencies such as difficulties in inhibiting voluntary responses, deficits in delaying gratification, and a low threshold for response). The extreme forms of impulsive behavior include aggressive or delinquent behaviors, substance dependence, and suicide attempts (Askénazy et al., 2003).

Previous research on the relationship between anxiety and impulsive behavior has resulted in mixed findings. One the one hand, anxiety and impulsivity have been traditionally considered to be orthogonally or inversely related (Barratt, 1965). Most notably, the reinforcement sensitivity theory proposed by Gray (1970, 1987) suggests that anxiety and impulsivity are two independent biologically based dimensions of personality, corresponding to avoidance and approach motivations, respectively (see also in Caci et al., 1998; Corr, 2002). Researchers have also pointed out that the characteristic features of anxiety such as behavioral inhibition and safety-seeking may be inconsistent with impulsivity (Taylor et al., 2008). On the other hand, it has been found that impulsive behavior could be caused by increased arousal and reduced cognitive efficiency (Del Carlo et al., 2012). Consistent with this idea, many studies have reported a link between anxiety and impulsivity (Jakuszkowiak-Wojten et al., 2015). For instance, Taylor et al. (2008) found that patients with a comorbid anxiety disorder showed higher levels of impulsivity compared to patients without an anxiety disorder (see also in Perugi et al., 2011; Del Carlo et al., 2012). To sum up, as pointed out by Askénazy et al. (2003), the relationship between anxiety and impulsivity is still highly controversial. In our opinion, it is largely because of the two conflicting characteristics of anxiety, i.e., an elevated level of physiological arousal (which may lead to approach behavior) and an excessive focus on threat-related stimuli (which may lead to avoidance behavior). Therefore, to investigate the relationship between anxiety and impulsivity could help understand the predominant motivation of anxious individuals and further unravel the psychological mechanisms of anxiety.

A potential reason for the heterogeneous findings in the literature is that most studies rely on self-report measures; however the validity of this method is limited by response bias, socially desirable responses, and participants' ability to provide accurate information (Fowles, 1987; Crowley et al., 2009). Regarding that, this study applies an experimental paradigm of delay discounting, which is widely considered as a behavioral performance measurement of impulsivity (Madden and Bickel, 2010). Delay discounting (also known as temporal discounting or time discounting) refers to a psychological phenomenon that outcomes decrease in value as a function of delay (Reynolds, 2006). During intertemporal decision-making, the delay discounting effect may manifest as a preference for the sooner but smaller monetary rewards over the larger delayed rewards (McClure et al., 2004). The results from the delay discounting paradigm have been interpreted to reflect impulsivity (Crean et al., 2000). To our knowledge, Rounds et al. (2007) first discovered that participants with high social anxiety showed a larger effect of delay discounting (but see Jenks and Lawyer, 2015). This finding was then extended by Zhao et al. (2015), who found that the relationship between anxiety and impulsive choices is not specific to social anxiety.

The current study aims to investigate the neural underpinning of delay discounting in anxiety, so as to enrich the understanding of the relationship between anxiety and impulsivity. We chose the event-related potential (ERP) technique for its exquisite temporal resolution (Amodio et al., 2014). Three ERP components have been associated with impulsivity and/or delay discounting, and based on which we compared the ERP differences between individuals with high and low trait anxiety (HTA and LTA). The first component is the N1, which is an early sensory component involved in selective attention and attention processing for goal-directed actions (Schupp et al., 2007; Baldauf and Deubel, 2009). Impulsive individuals have a larger N1 in response to visual stimuli, indicating enhanced attentional orienting compared to less impulsive individuals (Houston and Stanford, 2001). The second component is the reward positivity (RewP), which is traditionally known as the feedback error-related negativity and has been considered as a negative-going component (Gehring and Willoughby, 2002; Yeung and Sanfey, 2004). However, recent studies have revealed that it actually reflects reward-related dopaminergic activity and should be re-interpreted as a larger positivity in the positive feedback condition rather than a negative component in the negative feedback condition (Foti et al., 2011; Walsh and Anderson, 2012; Proudfit, 2015). In the delay discounting task, Cherniawsky and Holroyd (2013) found that a larger RewP elicited by immediate compared to delayed rewards indicates a stronger preference for impulsive choices (Onoda et al., 2010; Mason et al., 2012). In addition, the RewP might also reflect individual differences in intolerance of uncertainty, since this component is sensitive to the uncertainty of an outcome feedback (Hirsh and Inzlicht, 2008; Nelson et al., 2016). Finally, the P3 is regarded as a measure of motivational significance in the decision-making literature, i.e., reflecting the potential impact of an outcome feedback on levels of motivation (Yeung and Sanfey, 2004; Nieuwenhuis et al., 2005; Wu and Zhou, 2009). An enhanced P3 has been found in individuals who show a larger delay discounting effect, which may indicate stronger motivations to pursue immediate over delayed rewards (Li et al., 2012). Both the RewP and the P3 are the most important indexes of feedback processing during decision-making (San Martín, 2012).

Given previous research about delay discounting in anxiety, as well as characteristics of the ERP components described above, we predicted that: (1) on the behavioral level, the HTA group would exhibit an immediacy bias for rewards, indicating higher levels of impulsivity compared to the LTA group; (2) accordingly on the electrophysiological level, the LTA group would show larger N1 (reflecting a higher selective attention), RewP (reflecting a higher level of reward evaluation), and P3 amplitudes (reflecting a stronger motivation) for immediate choices, compared to the LTA group. In contrast, the HTA group would show higher P3 amplitudes for delayed choices. These findings would provide valuable knowledge about the underlying mechanism of the delay discounting bias in anxious people.

## Methods

### Participants

In view of the fact that anxiety and depressive symptoms are highly comorbid (Nieuwenhuis et al., 2005; Hirsh and Inzlicht, 2008; Nelson et al., 2016) and depressive patients were also impulsive in the delay discounting task (Wu and Zhou, 2009), we only recruited non-depressed participants with high trait anxiety (HTA) and non-depressed participants with low trait anxiety in this study.

All the freshman students (*n* = 6725) in Shenzhen University were required to complete the Trait form of Spielberger's State-Trait Anxiety Inventory (STAI-T; Spielberger et al., 1983; Shek, 1993). In this sample, individuals with STAI-T scores in the upper and lower 25% of the distribution were considered as HTA and LTA subjects, respectively (Gu et al., 2010; Luo et al., 2014). The Beck Depression Inventory Second Edition (BDI-II; Beck et al., 1996) was used to assess self-reported symptoms of depression. Only the participants with BDI-II scores <13 were considered in this study (Note: while BDI-II <13 indicates minimal depression, BDI-II ≥ 14 indicates mild, moderate, or severe depression; see Beck et al., 1996). From those who met these criteria, we randomly recruited 52 students as paid participants (26 in LTA group and 26 in HTA group^1^). There was no significant difference between the two groups with respect to age, handedness and BDI-II scores (Table 1).

**Table 1:** **Demographic data of HTA and LTA participants**.

**Characteristics**	**LTA (*n* = 26)**	**HTA (*n* = 26)**	**Statistics**
Mean age, y	19.4 (18–20)	19.7 (17–21)	*t*_(50)_ = −1.77, *p* = 0.083
Sex, male/female	13/13	13/13	
Handedness, right/left	26/0	26/0	
STAI-T	30.5 (20–43)	56.54 (51–70)	*t*_(50)_ = −15.13, *p* < 0.001
BDI-II	3.87(0–7)	4.96 (0–12)	*t*_(50)_ = −0.65, *p* = 0.185

*STAI-T, the Trait form of Spielberger's State-Trait Anxiety Inventory; BDI-II, Beck Depression Inventory (Second Edition). Descriptive data are presented as mean (range)*.

Exclusion criteria for both groups were (1) any Axis I and II disorders according to the Diagnostic and Statistical Manual (DSM-IV; APA, 1994); (2) seizure disorder; (3) history of head injury with possible neurological sequela, and (4) substance abuse or dependence in the past 6 months. These criteria were also designed to exclude the potential influence of psychiatric medications on the results (Onoda et al., 2010; Mason et al., 2012; Weisz et al., 2012; Needham et al., 2015).

Written informed consent was obtained prior to the experiment. The experimental protocol was approved by the Ethics Committee of Institute of Psychology, Chinese Academy of Sciences (H14019) and this study was performed strictly in accordance with the approved guidelines.

### Procedures

**Figure 1:**
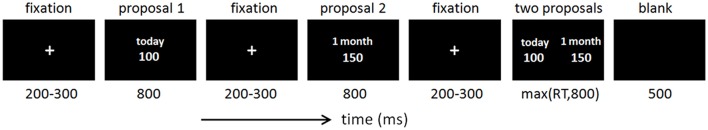
**Illustration of the delay discounting paradigm in this study**.

The experiment consisted of 480 trials. As shown in Figure 1, the “Proposal 1” was first displayed for 800 ms, with an earlier reward (today or 2 weeks later). Then the “Proposal 2” was displayed for 800 ms, with a delayed reward (2 weeks or a month later). Finally, the two proposals were presented simultaneously (the left and right sides of the two proposals were counterbalanced across trials) and subject was required to press the choice button as quickly as possible. There were three possible combinations of time delay in two proposals, i.e., “today” in Proposal 1 *vs*. “2 weeks later” in Proposal 2 (33.3%), “today” in Proposal 1 *vs*. “a month later” in Proposal 2 (33.3%), and “2 weeks later” in Proposal 1 *vs*. “a month later” in Proposal 2 (33.3%).

In each trial, participants were required to select from two options: one was with a shorter delay but a smaller reward (Proposal 1), and the other was with a longer delay but a larger reward (Proposal 2). The amount of money in “Proposal 1” was randomly (50 *vs*. 50%) chosen from two uniform distributions ([50, 80] and [100, 130]); the amount of money in “Proposal 2” was 10 and 50% (probability: 50 vs. 50%) higher than in “Proposal 1.”

Before the experiment, participants were told about the rules of the task and the meaning of the symbols. They were encouraged to respond according to their risk preference. Participants were also informed that they should consider every trial equally important, since a random trial would be selected at the end of the experiment and the chosen time delay in that trial would be the real delay for their monetary reward.

### Behavioral measures

In addition to reaction time, this study defined another behavioral measure, namely “impulsivity ratio,” to index the preference for impulsive decision-making in individuals.

We considered the selection of the immediate reward in Proposal 1 (a proposal with a shorter delay but a smaller reward) as an impulsive choice and the selection of the delayed reward in Proposal 2 (a proposal with a longer delay but a larger reward) as a non-impulsive choice. The tendency to choose the immediate reward indicates a preference for impulsive decision-making in intertemporal scenarios. This preference was measured as the “impulsivity ratio,” by dividing the number of impulsive choices (Proposal 1) by the total number of choices (Proposal 1 + Proposal 2). It was unnecessary to calculate the “non-impulsivity ratio,” because its value was equal to one minus the impulsivity ratio in each condition.

### EEG recording and analysis

Brain electrical activity was recorded referentially against left mastoid and off-line re-referenced to the average of the left and right mastoids, by a 64-channel amplifier with a sampling frequency of 250 Hz (Brain Products, Gilching, Germany). Electroencephalography (EEG) data were collected with electrode impedances kept below 5 kΩ. Ocular artifacts were removed from EEGs using a regression procedure implemented in NeuroScan software (Scan 4.3).

The recorded EEG data were filtered (0.01–30 Hz) and segmented beginning 200 ms prior to the onset of “Proposal 1” and “Proposal 2.” This study did not analyze the ERP epochs evoked by the presentation of “two proposals” (i.e., the screen in which two proposals were presented simultaneously) because participants may have already assigned values to Proposal 1 and Proposal 2 prior to this time point (Lebreton et al., 2009). All epochs were baseline-corrected with respect to the mean voltage over the 200 ms preceding the onset of stimulus, followed by averaging in association with experimental conditions. Trials contaminated with large artifacts (peak-to-peak deflection exceeded ± 100 μV) were excluded from the averaging. As a result, 35 ± 16 trials and 24 ± 34 trials were rejected in each subject for Proposal 1 and Proposal 2, respectively. Trial numbers did not show significant difference between experimental conditions.

This study focused on the ERPs elicited by the immediate option (Proposal 1) and the delayed option (Proposal 2) in the two groups. We analyzed the average amplitudes of the three ERP components (N1, RewP and P3) across different sets of electrodes according to grand-mean ERP topographies and relevant literatures (Onoda et al., 2010; Blackburn et al., 2012; Mason et al., 2012; Cherniawsky and Holroyd, 2013). The N1 was measured as the average amplitude occurring 170–210 ms after the onset of proposal presentation at the electrode sites of O1, O2, PO7, PO8, P7, and P8 (Mason et al., 2012). The RewP was defined as the average amplitude occurring 250–350 ms after the onset of proposal presentation at the electrode sites of Fz, FCz, FC1, FC2, Cz, C1, and C2 (Holroyd et al., 2008; Mason et al., 2012). The P3 was defined as the average amplitude occurring 300–450 ms after the onset of proposal presentation at the electrode sites of Pz, P3, P4, CPz, CP3, and CP4 (Wu et al., 2016).

### Statistics

Descriptive data were presented as mean ± standard error. The significance level was set at 0.05.

First, the behavioral measures (impulsivity ratio and response time) were analyzed using a two-way ANOVA, with choice (immediate *vs*. delayed choice) as the within-subject factor, and group (HTA *vs*. LTA) as the between-subject factor.

Then the ERP components time-locked to the Proposal 1 and Proposal 2 were analyzed separately. For the ERPs time-locked to the Proposal 1, a two-way ANOVA was used, with time delay (“today” and “2 weeks later”) as the within-subject factor, and group as the between-subject factor. For the ERPs time-locked to the Proposal 2, another two-way ANOVA was used, with choice (immediate *vs*. delayed choice) as the within-subject factor, and group as the between-subject factor.

Significant interactions were analyzed using simple effects model. *Post-hoc* testing of significant main effects was conducted using the Bonferroni method.

Finally, two-tailed Pearson's *r* correlation was performed between behavioral and ERP measurements. Correction for multiple comparisons was based on Holm's stepwise method.

## Results

In this section, we first report the behavioral results. Then the ERP results of the three components were reported. Finally, the correlation between behavioral and ERP measures were reported. For the sake of brevity, the experimental effects that did not reach significance were omitted.

### Behaviors

This study analyzed two behavioral measures, i.e., impulsivity ratio and response time.

#### Impulsivity ratio

**Figure 2:**
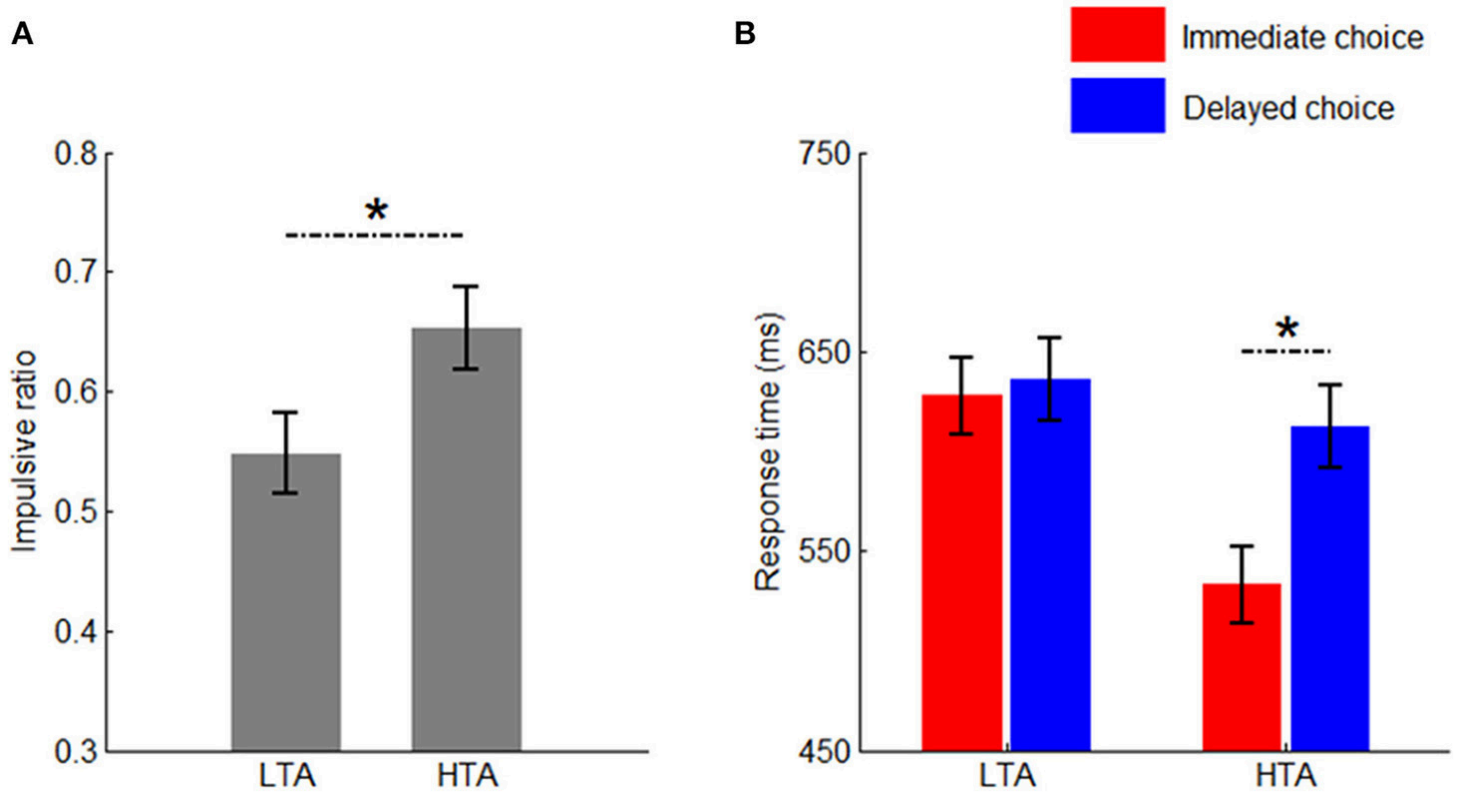
**Behavioral results. (A)** The impulsivity ratio in the two groups; **(B)** The response time in the two groups between immediate and delayed choices. Bars represent standard error of the mean. LTA, the low-trait anxiety group; HTA, the high-trait anxiety group. ^*^p < 0.05.

The main effect of group was significant [*F*_(1, 50)_ = 4.75, *p* = 0.034, $\eta_{p}^{2}$ = 0.087]. Compared with the LTA group (54.8 ± 3.4%), the HTA group made more impulsive choices (65.3 ± 3.4%; Figure 2A).

#### Response time (RT)

The main effect of group was significant [*F*_(1, 50)_ = 7.20, *p* = 0.010, $\eta_{p}^{2}$ = 0.126]. The HTA group (572 ± 15.7 ms) responded much faster than the LTA group did (632 ± 15.7 ms).

The main effect of choice was significant [*F*_(1, 50)_ = 5.97, *p* = 0.017, $\eta_{\text{p}}^{2}$ = 0.107]. The response time for the immediate choice (581 ± 13.6 ms) was significantly shorter than that for the delayed choice (624 ± 14.7 ms).

The interaction between choice and group was significant [*F*_(1, 50)_ = 4.07, *p* = 0.049, $\eta_{\text{p}}^{2}$ = 0.075; Figure 2B]. Compared with the LTA group (628 ± 19.2 ms), the HTA group responded faster when the immediate option was chosen (533 ± 19.2 ms). However, no significant difference was found between the two groups when the delayed option was chosen [*F*_(1, 50)_ < 1; LTA = 636 ± 20.8 ms, HTA = 612 ± 20.8 ms].

### ERPs

In this subsection, we first report the ERP results time-locked to the Proposal 1. Then the ERP results time-locked to the Proposal 2 were reported. The three ERP components were presented in a temporal sequence. For the sake of brevity, the experimental effects that did not reach significance were omitted.

#### ERP components evoked by the immediate option (proposal 1)

##### N1

**Figure 3:**
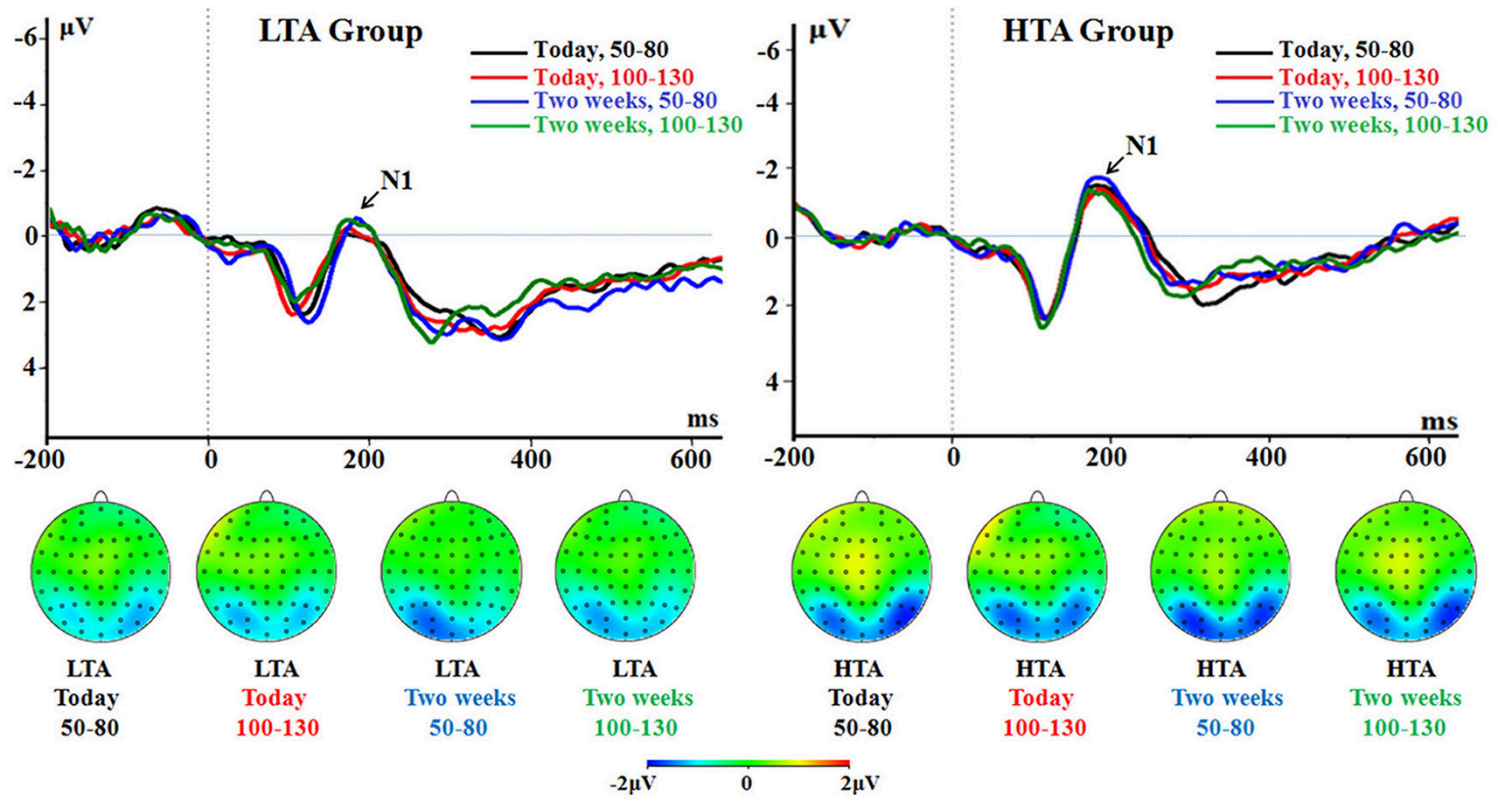
**The N1 component time-locked to the immediate option (Proposal 1)**. ERPs were calculated by averaging the data at the electrodes of O1, O2, PO7, PO8, P7, and P8.

The main effect of group was significant [*F*_(1, 50)_ = 5.75, *p* = 0.020, $\eta_{\text{p}}^{2}$ = 0.103]. The HTA group had a larger N1 (−1.12 ± 0.23 μV) compared with the LTA group (−0.36 ± 0.23 μV; Figure 3).

##### P3

**Figure 4:**
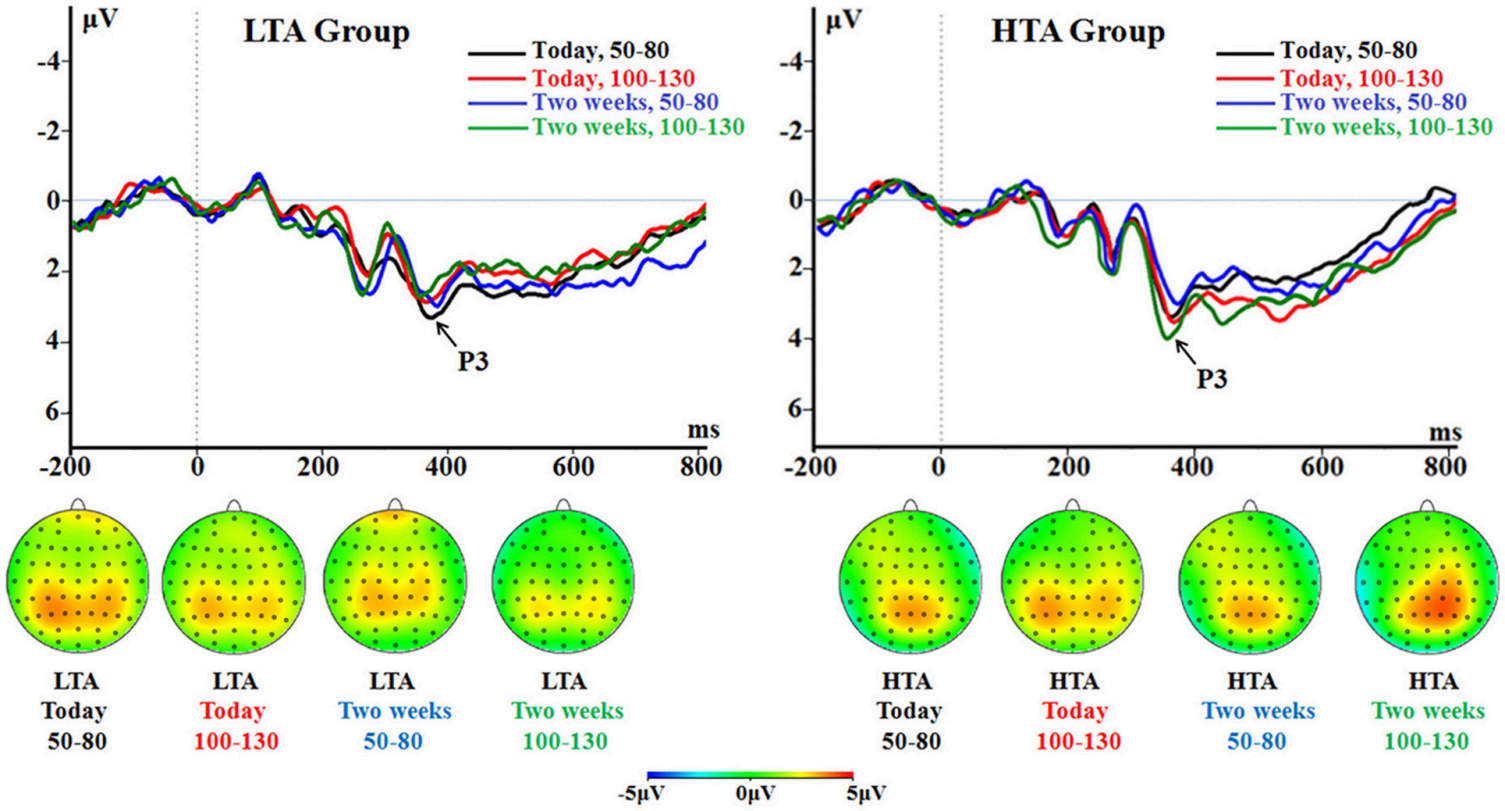
**The P3 component time-locked to the immediate option (Proposal 1)**. ERPs were calculated by averaging the data at the electrodes of Pz, P3, P4, CPz, CP3, and CP4.

The main effect of group was marginally significant [*F*_(1, 50)_ = 4.03, *p* = 0.050, $\eta_{\text{p}}^{2}$ = 0.075]; the HTA group had a larger P3 (2.88 ± 0.18 μV) compared with LTA group (2.39 ± 0.18 μV; Figure 4).

#### ERP components evoked by the delayed option (proposal 2)

##### N1

The main effect of choice was significant [*F*_(1, 50)_ = 8.69, *p* = 0.005, $\eta_{\text{p}}^{2}$ = 0.148]; the immediate choice evoked significantly larger N1 (−1.77 ± 0.23 μV) compared with the delayed choice (−1.11 ± 0.20 μV).

**Figure 5:**
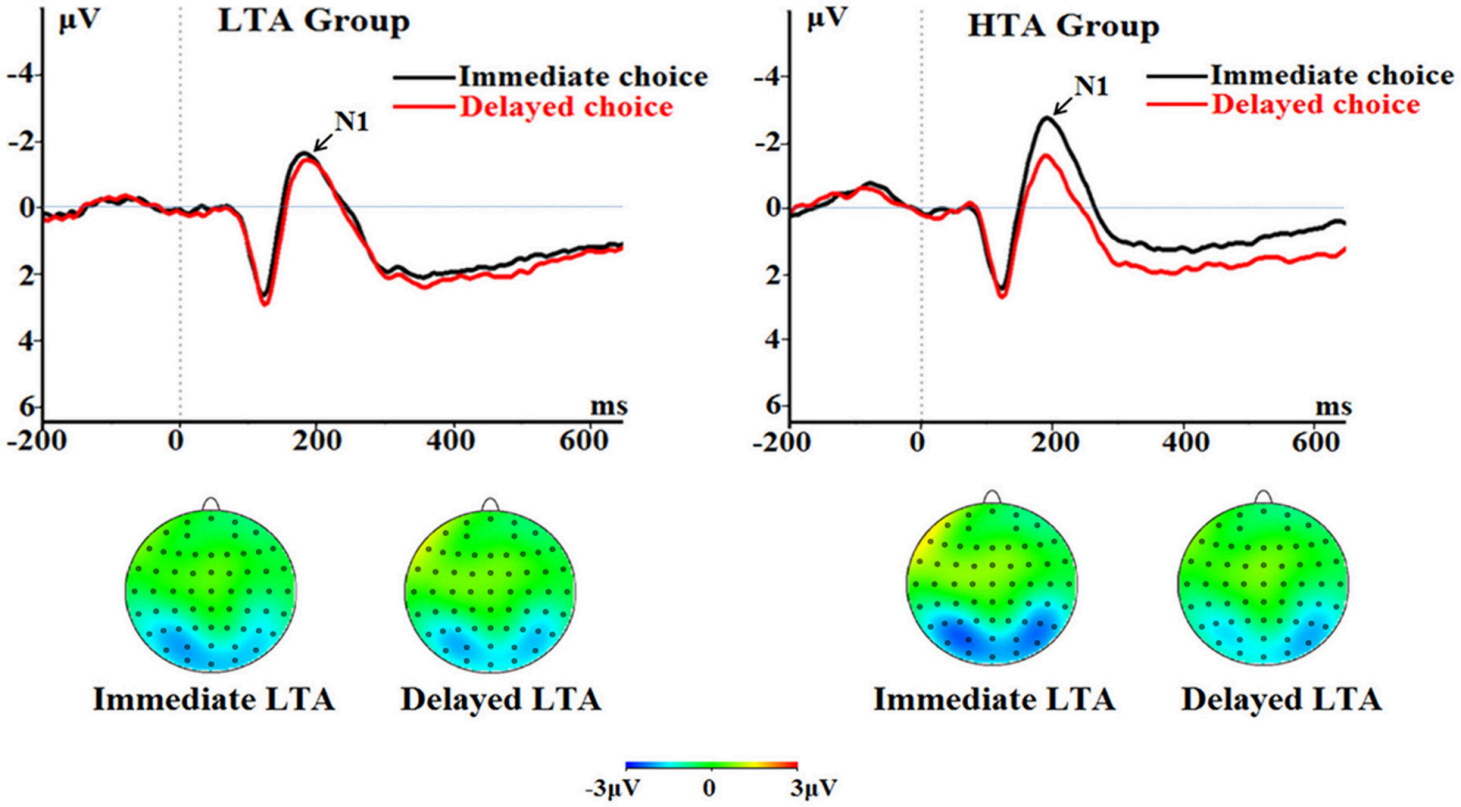
**The N1 component time-locked to the delayed option (Proposal 2)**. ERPs were calculated by averaging the data at the electrodes of O1, O2, PO7, PO8, P7, and P8.

The interaction between choice and group was significant [*F*_(1, 50)_ = 5.64, *p* = 0.021, $\eta_{\text{p}}^{2}$ = 0.101; Figure 5]. The immediate choice evoked a larger N1 (−2.23 ± 0.33 μV) in the HTA group than in the LTA group [−1.30 ± 0.33 μV; marginally significant, *F*_(1, 50)_ = 3.93, *p* = 0.053]. However, no group difference was found for the delayed choice [*F*_(1, 50)_ < 1; LTA = −1.18 ± 0.28 μV, HTA = −1.04 ± 0.28 μV].

##### RewP

The main effect of choice was significant [*F*_(1, 50)_ = 13.3, *p* = 0.001, $\eta_{\text{p}}^{2}$ = 0.210]. The immediate choice evoked a larger RewP (2.18 ± 0.29 μV) compared with the delayed choice did (1.47 ± 0.26 μV).

The main effect of groups was significant [*F*_(1, 50)_ = 4.90, *p* = 0.031, $\eta_{\text{p}}^{2}$ = 0.089]. The HTA group had a larger RewP (2.40 ± 0.37 μV) compared with the LTA group (1.26 ± 0.37 μV).

**Figure 6:**
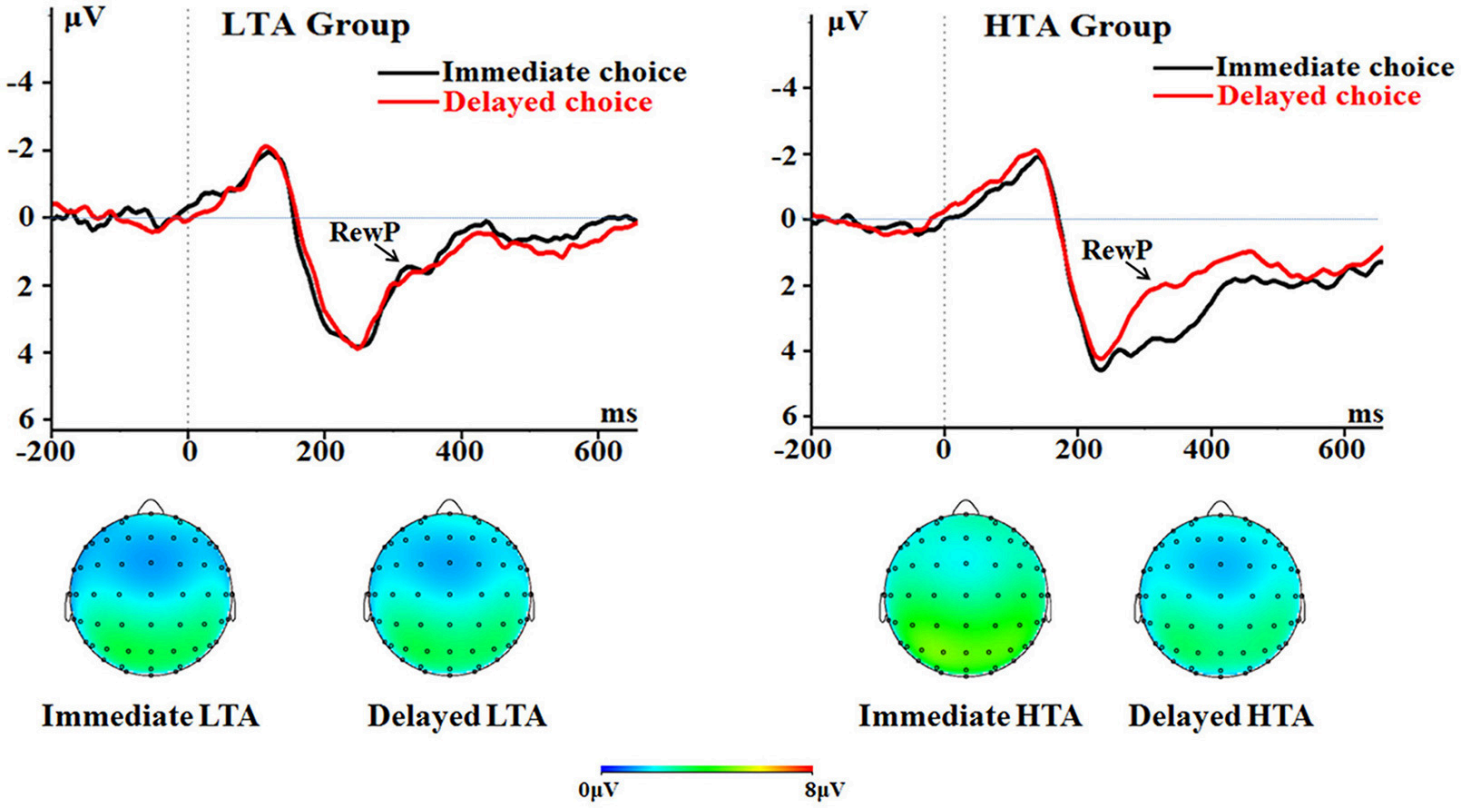
**The RewP component time-locked to the delayed option (Proposal 2)**. ERPs were calculated by averaging the data at the electrodes of Fz, FCz, FC1, FC2, Cz, C1, and C2.

The interaction between choice and group was significant [*F*_(1, 50)_ = 14.3, *p* < 0.001, $\eta_{\text{p}}^{2}$ = 0.222; Figure 6]. The immediate choice evoked a larger RewP (3.12 ± 0.41 μV) in the HTA group than in the LTA group [1.24 ± 0.41 μV; *F*_(1, 50)_ = 10.6, *p* = 0.002]. However, no group difference was found for the delayed choice [*F*_(1, 50)_ < 1; LTA = 1.27 ± 0.37 μV, HTA = 1.68 ± 0.37 μV].

##### P3

The main effect of choice was significant [*F*_(1, 50)_ = 14.1; *p* < 0.001; $\eta_{\text{p}}^{2}$ = 0.220]. The delayed choice evoked a larger P3 (2.85 ± 0.19 μV) compared with the immediate choice did (2.25 ± 0.22 μV).

**Figure 7:**
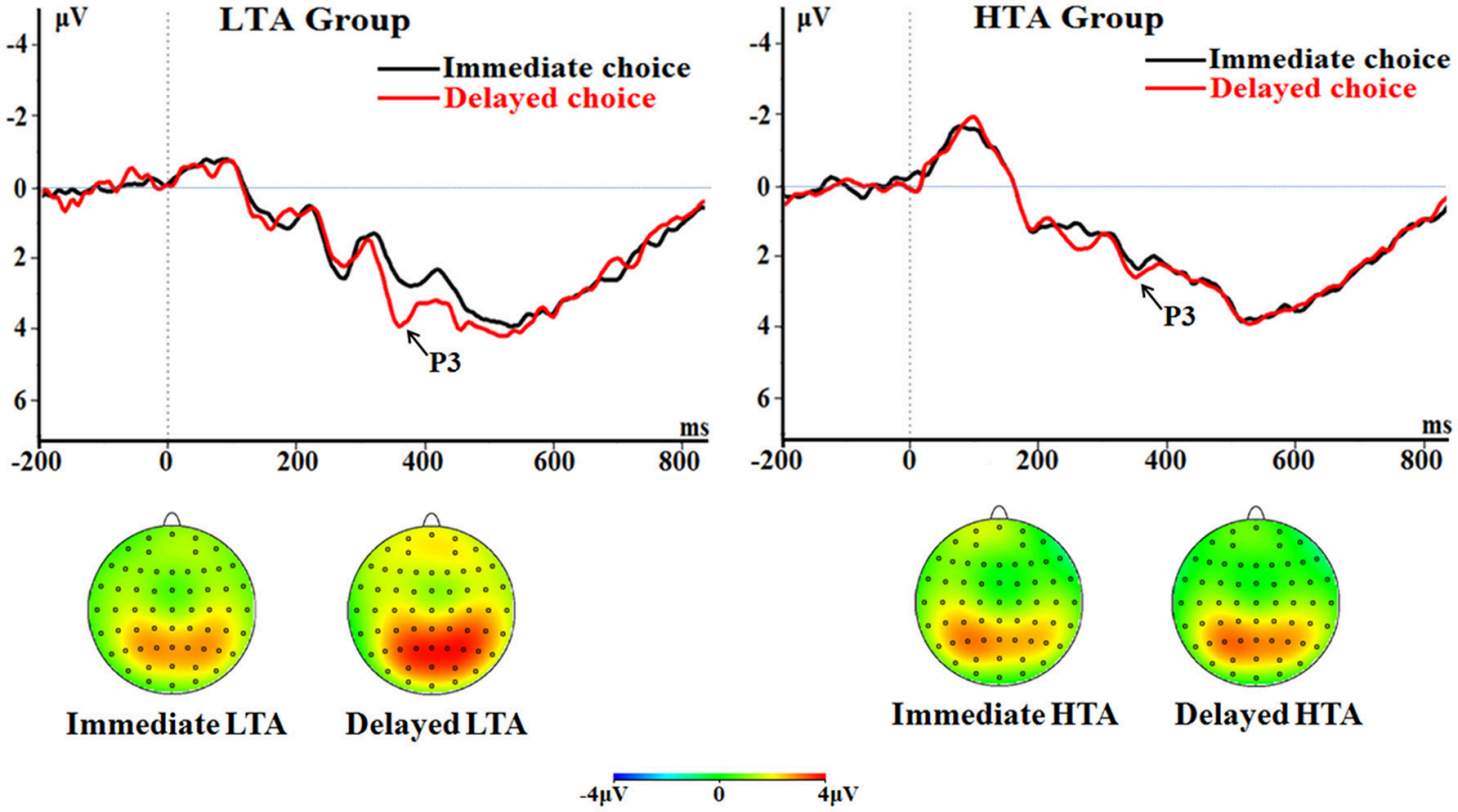
**The P3 component time-locked to the delayed option (Proposal 2)**. ERPs were calculated by averaging the data at the electrodes of Pz, P3, P4, CPz, CP3, and CP4.

The interaction between choice and group was significant [*F*_(1, 50)_ = 11.0; *p* = 0.002; $\eta_{\text{p}}^{2}$ = 0.180; Figure 7]. The delayed choice evoked a larger P3 (3.43 ± 0.27 μV) in the LTA group than in the HTA group [2.26 ± 0.27 μV; *F*_(1, 50)_ = 9.57, *p* = 0.003]. However, no group difference was found for the immediate choice [*F*_(1, 50)_ < 1; LTA = 2.30 ± 0.31 μV, HTA = 2.19 ± 0.31 μV].

### The correlation between behavioral and ERPs

According to the ERP results reported above, we conducted Pearson correlation analyses between the ERP measures (the three indexes which showed interaction between choice and group) and the RT (the only behavioral index which showed interaction between choice and group).

For the N1 and RewP evoked by the delayed option (i.e., the Proposal 2), we used the associated amplitudes in the condition when the immediate choice was selected. For the P3 evoked by the delayed option we used the amplitudes in the condition when the delayed choice was selected. For the RT, we used the data in the condition when the immediate choice was selected.

Totally three corrections were performed, resulting in two significant correlations after correction for multiple comparisons. The RewP (*r* = −0.37, *p* = 0.007, corrected *p* = 0.014) and P3 (*r* = 0.385, *p* = 0.005, corrected *p* = 0.015) were correlated significantly with the RT.

## Discussion

Debate exists on the relationship between trait anxiety and impulsive behavior. This study applied the delay discounting paradigm to compare the tendency of making impulsive choices between HTA and LTA participants. On the behavioral level, the HTA group chose the immediate option more often than the LTA group regardless of its incentive value. Additionally, the speed needed for choosing the immediate option was shorter in the HTA group than in the LTA group, while this RT difference was not significant when the delayed option was chosen.

According to the literature, we speculate that the delay discounting effect in anxious individuals is driven by their intolerance of uncertainty (IU; MacDonald et al., 2015). The term IU describes negative beliefs about future-oriented uncertainty, which is also a key feature of anxiety (Duronto et al., 2005; Maner and Schmidt, 2006; Bensi and Giusberti, 2007; Grupe and Nitschke, 2013). Patients diagnosed with anxiety disorder often show a higher IU score than the controls, possibly due to insufficient perception of personal control (Krain et al., 2008). Therefore, anxious individuals are more likely to underestimate the value of delayed rewards. In the current study, both the impulsivity ratio and the response time indicate that HTA participants favored immediacy. We suggest these behavioral results are consistent with previous findings that anxious individuals display high level of IU (Krain et al., 2008). That is to say, HTA participants avoided the delayed option because its association with uncertainty elicits a feeling of worry. However, a non-negligible limitation of the current study is that we did not include any behavioral measure of the IU level, such as the Intolerance of Uncertainty Scale (Buhr and Dugas, 2002). Follow-up research is necessary to address this issue directly.

On the electrophysiological level, three ERP components (N1, RewP, and P3) evoked by both the immediate option (Proposal 1) and the delayed option (Proposal 2) were analyzed. Previous literature suggest that during decision-making, the N1, RewP, and P3 reflect the processes of attentional orientation, encoding of reward value, and motivational evaluation, respectively (Polezzi et al., 2010; Blackburn et al., 2012; San Martín, 2012). The theoretical significance of the current ERP findings is interpreted under this framework.

First, the increased N1 amplitudes have been associated with high levels of impulsivity. For example, impulsive-aggressive participants exhibited a larger N1 in response to visual stimuli, indicating an enhanced attentional orientation (Gehring and Willoughby, 2002). In the current study, the N1 elicited by the immediate option was enhanced in the HTA group than in the LTA group, indicating that HTA participants paid more attention to this option. When participants' decisions (immediate vs. delayed) was taken into account, a significant choice by group interaction shows that the N1 became larger when HTA participants made an immediate decision, which also indicates more attentional resources being allocated. In our opinion, this result could be regarded as evidences that early attentional orientation contributes to anxious people's impulsive choices. As pointed out by Blackburn et al. (2012), impulsive decisions might be initially driven by an attentional bias toward immediate reward, which manifests as an enlarged N1 component. Therefore, the N1 finding indicates that the relationship between trait anxiety and impulsive choices is mediated by an attention allocation strategy that prefers immediacy at the early stage of option assessment. In line with our interpretation, previous studies using clinical assessments have discovered a positive correlation between trait anxiety symptoms and attentional impulsivity score in patients with anxiety disorder (Summerfeldt et al., 2004; Perugi et al., 2011).

Second, the RewP has been widely considered to represent the encoding of reward values (Lukie et al., 2014; Proudfit, 2015). Consistent with this classical theory, both immediate and delayed options evoked the RewP sensitive to the amount of reward. Most importantly, the RewP elicited by the delayed option showed a significant choice by group interaction, which was similar with the N1 pattern. That is, the RewP was larger when it was followed by an immediate decision in the HTA group than in the LTA group, but this effect was absent for the delayed decision. Seeing that the group difference selectively appeared on the RewP elicited by the Proposal 2 (the delayed option), we suggest that the RewP finding indicates that trait anxiety modulates the comparison of reward values between the two options. In line with this hypothesis, previous studies have reported that the RewP amplitude reflects the relative rather than absolute values of ongoing events (Holroyd and Coles, 2008). In our opinion, high levels of trait anxiety resulted in overestimation of the reward value for immediate options compared to delayed options; therefore the RewP was larger for the immediate choice than the delayed choice in HTA participants. In addition, recent studies have associated the RewP with individual difference in IU (Nelson et al., 2016). Therefore, the RewP finding could be regarded as supporting evidence that HTA individuals manifest higher levels of IU than their LTA counterparts.

Third, the P3 component is supposed to index the motivational significance of different options. Specifically, the P3 elicited by the immediate option was larger in the HTA group than in the LTA group. In addition, the P3 elicited by the delayed option was enhanced in the delayed decision condition for LTA compared to HTA participants, indicating that LTA participants had stronger motivations to select the delayed option. Taken together, the P3 finding reveals that the motivation level of HTA participants was more susceptible to the immediate option than the delayed option, which may help to explain their behavioral preference.

Finally, two limitations should be pointed out for an appropriate interpretation of the current result. First, this study only measured the level of trait anxiety in a healthy population. Seeing that the healthy individuals with high anxiety and the patients with anxiety disorders are qualitatively different (Belzung and Griebel, 2001), the generalizability of the current findings still await to be investigated in clinical populations. Second, similar with previous studies (San Martín, 2012; Wu et al., 2016), the temporal order of the immediate option and the delayed option was fixed, so as to help participants to reduce cognitive load and focus their attentions on decision-related information. Seeing that the event sequence modulates the characteristics of ERPs (e.g., the studies by Gu et al., 2011; Osinsky et al., 2012), future studies should apply an alternative temporal order to examine the robustness of our findings.

To sum up, this study has revealed that HTA participants made more impulsive decisions in the delayed discounting paradigm, which demonstrates a positive relationship between trait anxiety and impulsive behavior. In addition, the ERP results (including the N1, RewP, and P3) indicate that the psychological processes of attentional orientation, encoding of reward values, and motivational evaluation contribute to this phenomenon. Specifically, HTA individuals' preference for impulsive choices is the consequence of an enhanced attentional orientation to the immediate option, overvaluation of immediate rewards, and higher level of motivations associated with immediacy.

## Author contributions

RG and DZ designed the study; LX conducted the experiment; LX and DZ analyzed the data; LX, RG, DZ, and YL contributed to the manuscript.

### Conflict of interest statement

The authors declare that the research was conducted in the absence of any commercial or financial relationships that could be construed as a potential conflict of interest.

## Footnotes

^1^The sample size of this study was decided based on our previous studies (Wu et al., 2013; Xu et al., 2013), which also employed ERP method to examine decision-making issues in college students with trait anxiety. Furthermore, because small sample size undermines the reliability of results, scholars now recommend a large sample size in neuroscience studies (Button et al., 2013). Thus a relatively large sample size was used in this study.
